# Tuberculous otitis media: Clinical challenges and long-term complications in three cases

**DOI:** 10.1016/j.idcr.2025.e02483

**Published:** 2025-12-26

**Authors:** Marta Anioł-Borkowska, Aleksandra Niemczyk, Marcin Masalski, Krzysztof Morawski

**Affiliations:** a Department of Otolaryngology with the Pediatric Otolaryngology Unit, University Clinical Hospital in Opole, Opole, Poland; b Faculty of Health Sciences, University of Opole, Poland

**Keywords:** Tuberculous otitis media, Extrapulmonary tuberculosis, Middle ear infection, Chronic otorrhea, Facial nerve palsy, Tympanic membrane perforation

## Abstract

Tuberculous otitis media (TOM) represents a rare extrapulmonary manifestation of tuberculosis, typically presenting with nonspecific early symptoms that make diagnosis challenging. The aim of this report is to present three cases of TOM and to review their clinical manifestations, diagnostic work-up, therapeutic management, and complications. We describe two patients with isolated TOM and a third who developed pulmonary tuberculosis during the course of the disease. In all three cases, symptoms followed a similar progression: Eustachian tube dysfunction, conductive hearing loss, and recurrent, painless otorrhea refractory to antibiotic therapy, accompanied by tympanic membrane (TM) perforations. Facial nerve palsy (FP) developed in two patients, either as a complication of untreated infection or following antromastoidectomy. Household exposure to *Mycobacterium tuberculosis* was confirmed in two of the three patients. The time from symptom onset to diagnosis was 6, 11, and 14 months, with the shortest delay observed in the patient who developed concurrent pulmonary symptoms. All patients received the standard four-drug regimen recommended by the WHO. Despite treatment, permanent complications persisted, including hearing loss, TM perforations, FP, postauricular fistula, and skin defects of the external auditory canal. These cases underscore that recurrent, painless otorrhea unresponsive to antibiotic therapy should prompt evaluation for TOM, particularly when accompanied by FP. Intraoperative identification of necrotic tissue in such cases warrants not only histopathological examination but also smear microscopy for acid-fast bacilli (AFB). The nonspecific clinical presentation and rarity of TOM contribute to initial misdiagnoses, leading to delays in establishing the correct diagnosis and initiating appropriate treatment.

## Introduction

Tuberculosis (TB) is a global health concern and remains the leading cause of death from infectious diseases worldwide [1]. In approximately 80 % of cases, the disease affects the lungs. The remaining 20 % represent extrapulmonary tuberculosis most commonly involving the lymph nodes, bones and joints, genitourinary system, meninges, and less frequently, the organs of vision and hearing [2], [3], [4]. Tuberculous otitis media (TOM) is a rare form of extrapulmonary tuberculosis, accounting for approximately 0.1 % of all tuberculosis cases [5] and 0.04–0.9 % of all chronic suppurative middle ear diseases [6], [7], [8].

Common initial symptoms of TOM, such as conductive hearing loss, painless otorrhea, and tympanic membrane (TM) perforations, as well as facial nerve palsy (FP), which may develop later in the course of the disease, are not distinctive and resemble those of other forms of chronic middle ear conditions [5], [7], [9], [10], [11], [12], [13], [14], [15]. The diagnosis is often considered only after additional findings appear, such as pulmonary symptoms [9], [10], [11], caseous granulomas [8] or mastoid abscesses [9], [14].

When TOM is suspected, bacteriological confirmation should always be pursued. Acid-fast bacilli (AFB) may be detected in ear swabs or histopathological specimens stained using the Ziehl–Neelsen method. Definitive diagnosis of infection with *Mycobacterium tuberculosis* complex requires molecular testing or culture, the latter being the diagnostic gold standard and allowing for drug susceptibility testing [16].

The low prevalence of TOM, its nonspecific symptoms, and the resulting diagnostic challenges often lead to delayed diagnosis, which may result in irreversible complications. Increasing awareness of this form of tuberculosis among healthcare professionals is therefore essential.

## Case reports

We present three immunocompetent Polish patients without socioeconomic deprivation and with adequate living conditions who were initially treated for chronic otitis media.

## Case 1

**Fig. 1:**
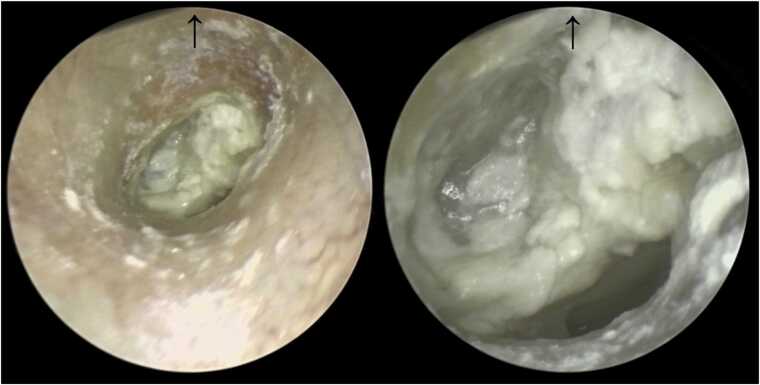
White, friable, avascular tissue filling the tympanic cavity in a patient with tuberculous otitis media (TOM), consistent with caseous granulation tissue. The tympanic membrane itself is not directly visible; the perforation is located in the anteroinferior quadrant, as confirmed by the Valsalva maneuver. Arrows indicate the superior (cranial) direction of the image.

A 34-year-old woman developed persistent right-sided aural fullness three weeks after septoplasty. Audiological testing demonstrated conductive hearing loss of up to 30 dB with a type B tympanogram. Standard treatment for Eustachian tube dysfunction, including cetirizine, pseudoephedrine, and intranasal corticosteroids proved ineffective. Four months later, spontaneous TM perforation was followed by recurrent painless otorrhea, with only transient improvement after topical and systemic antibiotics. After several months, she developed ipsilateral FP, classified as House–Brackmann (HB) grade IV. High-resolution computed tomography (HRCT) revealed opacification of the tympanic cavity and the mastoid air cells with preservation of the ossicular chain. The patient was hospitalized and treated with intravenous amoxicillin–clavulanic acid and systemic corticosteroids, which led to complete recovery of facial nerve function. After several weeks of recurrent otorrhea and eleven months after the onset of disease, the patient underwent canal wall-up antromastoidectomy. Intraoperatively, a white, friable, avascular tissue filling the tympanic cavity was observed (Fig. 1). Histopathological examination of the specimen obtained during antromastoidectomy revealed only necrosis, which did not allow for a conclusive diagnosis.

Two weeks postoperatively, she developed recurrent FP accompanied by otalgia, vertigo, and nausea, suggestive of labyrinthine involvement. She was re-hospitalized for further diagnostic evaluation and management. Cultures yielded *Staphylococcus epidermidis*. The QuantiFERON-TB test, an interferon-gamma release assay (IGRA) [17], was negative, and chest radiography was unremarkable. Despite intravenous ceftriaxone, topical ciprofloxacin, systemic corticosteroids, and vitamin B supplementation, FP progressed from grade II to grade V on the HB scale.

Re-antromastoidectomy was performed. Histopathological examination again demonstrated necrosis, this time accompanied by granulomatous inflammation and multinucleated giant cells, raising suspicion of TOM. Ziehl–Neelsen staining confirmed the presence of AFB. Direct ear smear was positive for AFB on microscopy, and culture confirmed growth of *Mycobacterium tuberculosis* complex, which was susceptible to all tested antituberculous drugs.

Standard four-drug therapy with isoniazid (H), rifampin (R), pyrazinamide (Z), and ethambutol (E) was administered for 3 months, followed by HR for an additional 9 months (3HRZE/9HR). The regimen was extended beyond the standard course (2HRZE/4HR) due to the persistent presence of numerous AFB in ear swabs, despite negative cultures. A detailed environmental history revealed that the patient’s partner had been treated for pulmonary tuberculosis (PTB) three years earlier. Sequelae of TOM included proximal external auditory canal skin defect, mesotympanic perforation, HB grade IV FP, and profound right-sided hearing loss. Additionally, the patient continues to experience non-tuberculous otorrhea in most episodes of upper respiratory tract infection, for which petrosectomy is being considered in the future.

## Case 2

A 29-year-old woman in her fourth week of pregnancy presented with nasal obstruction and persistent aural fullness lasting over a month after an upper respiratory tract infection treated with amoxicillin–clavulanate. Nasal endoscopy showed mucous discharge and a fibrinous coating near the right torus tubarius, while otoscopy revealed middle-ear effusion. Myringotomy was performed during hospitalization. Culture yielded *Pseudomonas aeruginosa*, and topical ciprofloxacin therapy led to local improvement and TM closure.

Right-sided otorrhea recurred several weeks later and, one week afterward, was followed by sinusitis and acute otitis media of the left ear. Culture from the right ear grew *Streptococcus pneumoniae*. The patient underwent sinus puncture and ventilation tube placement in the left ear. Systemic therapy with ceftazidime and piperacillin–tazobactam resulted in sterile cultures from both the ear and the sinuses. Despite the treatment, the patient developed a productive cough, and further empiric antibiotic therapy, including azithromycin, showed limited effectiveness.

Given the family history of pulmonary tuberculosis (her father and brother had been diagnosed three years earlier), evaluation for TB was undertaken. Sputum smear revealed AFB, with molecular testing and subsequent culture confirming *Mycobacterium tuberculosis* complex. Standard therapy (2HRZE/4HR) was initiated. During treatment, she delivered a full-term infant with congenital pneumonia of non-tuberculous etiology. The otologic, sinonasal, and respiratory symptoms underwent a gradual resolution, which, when considered in conjunction with the disease course, provided substantial evidence in support of a tuberculous etiology.

As a consequence of the disease, the patient developed bilateral subtotal tympanic membrane perforations with conductive hearing loss of approximately 40 dB HL. Over subsequent years, she underwent three right- and two left-sided myringoplasties, yet small bilateral perforations persisted. During nearly a decade of follow-up, no TB recurrence was noted, despite intermittent otorrhea usually associated with upper respiratory tract infections.

## Case 3

A 19-year-old woman with no significant medical history developed right-sided aural fullness and conductive hearing loss following intensive nasal irrigation performed for rhinosinusitis. Initial treatment for Eustachian tube dysfunction included cetirizine with pseudoephedrine and intranasal corticosteroids. Due to a lack of improvement, empirical antibiotic therapy with amoxicillin and cefuroxime was introduced, but proved ineffective. Endoscopy performed six weeks later revealed edema of the right torus tubarius and dried secretions in the nasopharyngeal roof. Audiological assessment showed a 40 dB conductive hearing loss with a type B tympanogram. In the following months, the patient noted increased sensation of ear moisture with progressive hearing loss, without otalgia. Further diagnostic work-up revealed a normal chest X-ray, negative QuantiFERON-TB test, and scant growth of *Staphylococcus* spp. in the right ear culture. HRCT demonstrated complete opacification of the tympanic cavity and mastoid air cells with an intact ossicular chain. Nine months after symptom onset, right-sided canal wall-up antromastoidectomy was performed.

**Fig. 2:**
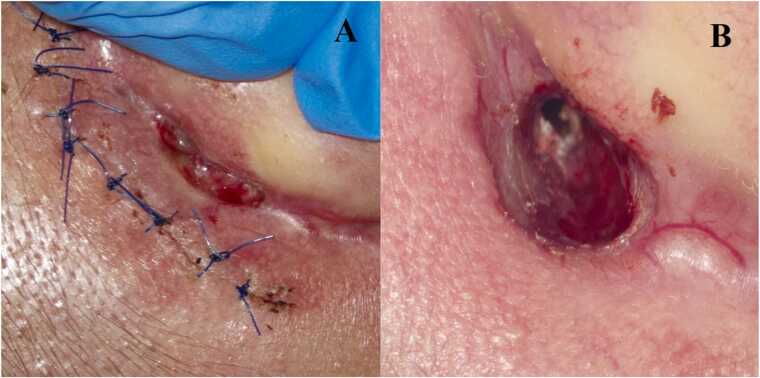
Development of a postauricular fistula in a patient with tuberculous otitis media (TOM). (A) Early stage of fistula formation at postoperative day 7, presenting as erythematous skin breakdown. (B) Status of the lesion during anti-tuberculous treatment, with marked reduction of inflammation and evidence of healing.

Approximately three weeks after surgery, the patient developed peripheral FP, HB grade IV. She was re-hospitalized and underwent revision surgery. During both ear surgeries, caseous granulation tissue was noted macroscopically and on histopathology corresponded to caseating necrosis with granulomatous inflammation and multinucleated giant cells, raising suspicion of TOM. Ziehl–Neelsen staining demonstrated AFB. Molecular testing identified *Mycobacterium tuberculosis* complex, and culture confirmed mycobacterial growth. During antituberculous treatment (2HRZE/4HR), gradual resolution of FP was observed. At the same time, a postauricular fistula developed, opening into the postoperative cavity (Fig. 2).

Three months after completion of antituberculous therapy, the patient underwent surgical closure of the postauricular fistula with tympanic membrane reconstruction. Facial nerve function improved to House–Brackmann grade II, but severe mixed hearing loss persisted.

## Discussion

This report presents three cases of TOM in young female patients aged 34, 29, and 19 years at disease onset. All were younger than the global mean age for TOM, estimated at 40 years [7]. Poor socioeconomic conditions typically associated with tuberculosis [7], [18] were not present in these patients.

In all three cases, TOM began with Eustachian tube dysfunction and otitis media with effusion, accompanied by conductive hearing loss and a type B tympanogram. Patient 1 developed symptoms after septoplasty, whereas Patients 2 and 3 presented nasopharyngeal abnormalities near the Eustachian tube orifice: a fibrinous coating (Patient 2) and mucosal edema (Patient 3). These findings support an ascending route of infection via the Eustachian tube. Additionally, after completion of anti-tuberculosis treatment, Patients 1 and 2 experienced recurrent non-tuberculous otorrhea during upper respiratory tract infections, suggesting persistent Eustachian tube and middle ear dysfunction following TOM.

Environmental exposure to *Mycobacterium tuberculosis* is a known risk factor for TOM [7], identified in two of the three cases. Patient 1’s partner had been treated for PTB three years prior to her symptom onset, while Patient 2’s brother and father were diagnosed with PTB during her evaluation for chronic nasal and middle ear inflammation. All three patients had received BCG (Bacillus Calmette–Guérin) vaccination in infancy, which primarily protects against severe forms of childhood TB but does not prevent infection. None of the patients showed immunosuppression or diabetes mellitus, both recognized risk factors for PTB [7].

All three patients experienced recurrent otorrhea typical of TOM, refractory to antibiotic therapy. In Patients 2 and 3 it was painless, whereas Patient 1 developed otalgia after surgical intervention. Painless otorrhea is the most common symptom of TOM, reported in approximately 90–100 % of cases [7], [19], [20]. Its diagnostic significance should be emphasized, as it may facilitate early implementation of targeted investigations for TB. Otalgia, by contrast, is less common [7].

All three patients initially presented with conductive hearing loss. Patient 1 and 3 later developed a sensorineural component. Reported TOM-related hearing loss ranges from 86 % to 100 % [7], [19], most often conductive (65.5 %), followed by mixed (31 %) and sensorineural (3.4 %) types [7].

The mean diagnostic delay for TOM reported in the literature is 12.8 months [7], consistent with our cases: 14 months in Patient 1, 11.5 in Patient 3, and 6 in Patient 2. In the latter, pulmonary symptoms prompted earlier evaluation. This highlights the diagnostic challenges of TOM, with faster recognition when PTB is present. Reports indicate that 25–50 % of ENT TB cases involve PTB [21], [22], [23].

QuantiFERON-TB testing was performed in two patients and yielded negative results in both. This finding confirms the limited diagnostic utility of the assay in isolated TOM [24].

FP occurred in two patients, resulting from untreated infection and as a postoperative complication of antromastoidectomy. This is consistent with reports indicating that TOM carries a higher risk of FP than other forms of chronic otitis media [7].

TOM is most commonly diagnosed postoperatively, accounting for 74–83.6 % of cases reported in the literature [7], [25]. This pattern was observed in Patients 1 and 3, in whom the diagnosis was first considered based on intraoperative tissue samples. These cases highlight the crucial role of histopathology and the pathologist in establishing the diagnosis. Although surgery provided definitive confirmation, it was followed by complications: FP in both patients, vestibular dysfunction in Patient 1, and a postauricular fistula in Patient 3.

In line with WHO recommendations for extrapulmonary TB [26], all three patients received standard four-drug anti-tuberculosis therapy (3HRZE/9HR in one case and 2HRZE/4HR in the other two), which resulted in pathogen eradication. Although surgery may aid diagnosis and therapy in TOM, pharmacological treatment remains the primary therapeutic approach.

## Conclusions

The presented cases and literature review indicate that the nonspecific, infrequent nature of tuberculous otitis media (TOM) often leads to initial misdiagnosis and delays in appropriate treatment. A characteristic chronological pattern of the disease was observed: in all patients, the earliest manifestations were related to Eustachian tube dysfunction, with otitis media with effusion and conductive hearing loss preceding the development of painless otorrhea refractory to conventional antibiotic therapy, frequently associated with tympanic membrane perforations. Prolonged, painless otorrhea unresponsive to standard treatment for bacterial otitis media, particularly when complicated by facial palsy, should therefore prompt targeted diagnostic evaluation for TOM, including bacteriological and molecular testing. Although surgical procedures may support the diagnostic process by providing tissue for histopathological and microbiological assessment, they are associated with an increased risk of complications in patients with TOM and must be considered cautiously. Finally, the persistence of non-tuberculous otorrhea during upper respiratory tract infections in two of the three patients following treatment suggests ongoing Eustachian tube and middle ear dysfunction, highlighting the need for long-term follow-up and functional monitoring after resolution of TOM.

## CRediT authorship contribution statement

**Marta Anioł-Borkowska:** Methodology, Investigation, Data curation, Writing – original draft, Writing – review & editing. **Aleksandra Niemczyk:** Methodology, Investigation, Writing – original draft, Writing – review & editing. **Marcin Masalski:** Conceptualization, Methodology, Investigation, Resources, Data curation, Writing – original draft, Writing – review & editing, Supervision. **Krzysztof Morawski:** Conceptualization, Resources, Writing – review & editing, Supervision.

## Patients consent

Written informed consent was obtained from the patients for publication of this case report and accompanying images. Copies of the written consent is available for review by the Editor-in-Chief of this journal on request.

## Declaration of Generative AI and AI-assisted technologies in the writing process

During the preparation of this work the authors used ChatGPT-4o and DeepL.com in order to translate the manuscript from Polish. After using these tools, the authors reviewed and edited the content as needed and take full responsibility for the content of the published article.

## Funding

No funding was received for the preparation of this manuscript.

## Declaration of Competing Interest

The authors declare that they have no known competing financial interests or personal relationships that could have appeared to influence the work reported in this paper.

## Data Availability

The data underlying this article are available from the authors on reasonable request, in accordance with applicable data protection regulations.

## References

[1] *Global Tuberculosis Report 2024*. World Health Organization; 2024. Accessed December 7, 2025. 〈https://www.who.int/publications/i/item/9789240101531〉. ISBN 978-92-4-010153-1.

[2] Fain O., Lortholary O., Lascaux V. (2000). Extrapulmonary tuberculosis in the northeastern suburbs of Paris: 141 cases. Eur J Intern Med.

[3] Baykan A.H., Sayiner H.S., Aydin E., Koc M., Inan I., Erturk S.M. (2022). Extrapulmonary tuberculosıs: an old but resurgent problem. Insights Imaging.

[4] Gopalaswamy R., Dusthackeer V.N.A., Kannayan S., Subbian S. (2021). Extrapulmonary tuberculosis—an update on the diagnosis, treatment and drug resistance. J Respir.

[5] Velepic M., Vukelic J., Dvojkovic Z., Skrobonja I., Braut T. (2021). Middle ear tuberculosis in an immunocompromised patient: case report and review of the literature. J Infect Public Health.

[6] Jeanes A.L., Friedmann I. (1960). Tuberculosis of the middle ear. Tubercle.

[7] Pai K.K., Omiunu A.O., Peddu D.K. (2022). Tuberculosis of the middle ear: a systematic review. Am J Otolaryngol Head Neck Med Surg W B Saunders.

[8] Scorpecci A., Bozzola E., Villani A., Marsella P. (2015). Two new cases of chronic tuberculous otomastoiditis in children. Acta Otorhinolaryngol Ital.

[9] Hajare P.S., Padmavathy O., Bellad S.A. (2022). Changing clinical scenario of tuberculous otitis media: a case series. Indian J Otolaryngol Head Neck Surg.

[10] Skolnik P.R., Nadol J.B., Sullivan Baker A. (1986). Tuberculosis of the middle ear: review of the literature with an instructive case report. Rev Infect Dis.

[11] Wallner L.J. (1953). Tuberculous otitis media. Laryngoscope.

[12] Djeric D., Tomanovic N., Boricic I. (2013). Tuberculous otitis media-diagnosis and treatment of four cases. J Int Adv Otol.

[13] Wadowski W., Brożek-Mądry E., Kawecki A., Krzeski A. (2022). Bilateral tuberculous otitis media – case report. Pol PrzegląD Otorynolaryngologiczny.

[14] Czesak M., Sokołowski J., Bartoszewicz R., Niemczyk K. (2015). Tuberculous otitis media - case presentation. Pol PrzegląD Otorynolaryngologiczny.

[15] Rękawek M., Kelar I., Radomska K. (2025). Tuberculous Otitis media complicated by bilateral deafness and bilateral nerve palsy – a case report. Pol PrzegląD Otorynolaryngologiczny.

[16] Augustynowicz-Kopeć E., Demkow U., Grzelewska-Rzymowska I. (2013). Guidelines of Polish Respiratory Society concerning diagnosis, treatment and prevention of tuberculosis in adults and in child. Pneumonol Alergol Pol.

[17] Sotgiu G., Saderi L., Petruccioli E. (2019). QuantiFERON TB Gold Plus for the diagnosis of tuberculosis: a systematic review and meta-analysis. J Infect.

[18] Hasan B., Hamdan Z., Mohammad A.N. (2025). A rare presentation of tuberculous otitis media in an immunocompetent adult: case report and literature review. Otolaryngol Case Rep.

[19] Aguilera-Franco M., Franco-Acosta A., Yépez-Naranjo A.F., Rodríguez-Granger J., Sampedro A., Navarro-Mari J.M. (2023). Tuberculous otitis media: a case presentation and review of european literature. Rev Esp De Quimioter.

[20] Sebastian S.K., Singhal A., Sharma A., Doloi P. (2020). Tuberculous otitis media –series of 10 cases. J Otol.

[21] Kaur J., Deshmukh P.T., Gaurkar S.S. (2024). Otorhinolaryngologic manifestations of tuberculosis: a comprehensive review of clinical and diagnostic challenges. Cureus.

[22] Michael R.C., Michael J.S. (2011). Tuberculosis in otorhinolaryngology: clinical presentation and diagnostic challenges. Int J Otolaryngol.

[23] Williams R.G., Douglas-Jones T. (1995). Mycobacterium marches back. J Laryngol Otol.

[24] Kim Y.J., Kang J.Y., Kim S., Il, Chang M.S., Kim Y.R. (2018). Predictors for false-negative QuantiFERON-TB Gold assay results in patients with extrapulmonary tuberculosis. BMC Infect Dis.

[25] Abes G.T., Abes F.L.L., Cruz T.L.G., Llanes E.G.D.V. (2023). Clinical spectrum of tuberculosis otitis Media (TBOM) and management outcomes. Acta Med Philos.

[26] WHO Operational Handbook on Tuberculosis*. Module 1: Prevention - Tuberculosis Preventive Treatment*. World Health Organization; 2024. Accessed December 7, 2025. https://www.who.int/publications/i/item/9789240097773. ISBN 978-92-4-009777-3.

